# A Synthetic Pathway for Producing Carbon Dots for Detecting Iron Ions Using a Fiber Optic Spectrometer

**DOI:** 10.3390/s25196066

**Published:** 2025-10-02

**Authors:** Ariana Adkisson, Dean Gouramanis, Ki-Joong Kim, Ward Burgess, Nicholas Siefert, Scott Crawford

**Affiliations:** 1 National Energy Technology Laboratory, 626 Cochran Mill Road, Pittsburgh, PA 15236, USA; aadkis5@lsu.edu (A.A.); ki-joong.kim@netl.doe.gov (K.-J.K.); ward.burgess@netl.doe.gov (W.B.); nicholas.siefert@netl.doe.gov (N.S.); 2 Fluid Photonics Corporation, Plainview, NY 11803, USA; dean@fluidphotonics.com; 3 NETL Support Contractor, 626 Cochran Mill Road, Pittsburgh, PA 15236, USA

**Keywords:** iron sensor, critical minerals, fiber optic, carbon dots, acid mine drainage

## Abstract

**Highlights:**

**What are the main findings?**

- Acid–base synthesis is employed as a convenient method for developing a low-cost sensor for iron ions, even in acidic, high ionic strength environments.
- A “low-cost” set-up using carbon dots with minimal processing and a compact, lab-built fiber optic spectrometer demonstrates low parts-per-million limits of detection for iron.

**What is the implication of the main finding?**

- Coupling low-cost fiber optic instrumentation with inexpensive yet highly sensitive carbon dot materials presents a promising avenue for environmental monitoring and process optimization.
- The synthesis of carbon dots through an acid–base approach may be used for the rational design of new metal ion sensors.

**Abstract:**

Iron detection is of growing importance in the critical minerals sector, where unwanted iron ions are typically removed during the processing of target critical metals. The ideal sensor should utilize inexpensive, scalable materials along with a low-cost, robust, and easy-to-use analysis platform. Here, we demonstrate a simple acid–base synthesis of luminescent iron-responsive carbon dots by reacting ethanolamine, phosphoric acid, and m-phenylenediamine. The carbon dots exhibit selective, iron-specific emission quenching, with the ability to detect part-per-billion levels of iron ions even in 0.1 M HCl. After benchmarking the purified materials using a commercial spectrometer, a “low-cost” process is demonstrated in which carbon dots with minimal purification are coupled with a portable fiber-optic spectrometer for analyzing iron content. Carbon dot-coated paper strips are also evaluated as another convenient platform for iron analysis. Taken together, the sensing material and platforms demonstrated here are well-suited for detecting trace quantities of iron in environmentally relevant conditions, with potential applications in tracking iron removal processes during critical mineral production as one exciting area of interest.

## 1. Introduction

Economically critical metals are vital to a range of important technologies, such as semiconductors, batteries, optical displays, and even missile defense systems [1,2]. However, the global supply chain for these metals faces significant instability risks due to a heavy reliance on just a handful of countries to meet demand [3]. Critical metals could be acquired from conventional mining practices, but the associated economic and environmental costs make this route increasingly challenging [4]. Consequently, there has been a growing interest in domestically sourcing critical metals from alternative sources, including electronic waste streams [5], produced waters [6], coal [7], and coal utilization byproducts, such as fly ash [8] and acid mine drainage (AMD) [9].

One of the key challenges for critical mineral production from both conventional and unconventional streams is the separation of target metals from non-target metals. Indeed, the removal of iron is often one of the first steps in processing critical metals from coal-based sources such as fly ash [10,11,12]. Thus, the ability to monitor iron removal in real time is critical for process optimization, maximizing efficiency, and minimizing costs [13]. Conventional characterization techniques used for iron analysis often require expensive equipment, such as inductively coupled plasma mass spectrometers (ICP-MS), which have instrumentation costs alone of over $100,000. Moreover, the lack of portability for these instruments prevents real-time analysis, creating time delays that can also increase cost. Thus, there is a critical need to develop sensing platforms for iron that are inexpensive and portable, while also having sufficient sensitivity to detect low ppm concentrations or better of target metals in complex environments (e.g., high ionic strength, low pH) typically encountered in metals processing streams [14,15]. Successful sensor development, therefore, requires innovation both in materials science and in platform development [16].

Carbon dots (CDs) are an exciting material for sensing economically critical metal ions due to their intense, tunable emission properties [17], photostability [18], ease-of-functionalization [19], and low cost (relative to, for example, metal nanoparticles) [20]. The design space for developing CD sensors is nearly inexhaustible; a wide variety of carbon precursors may be used [21], CDs may be doped with different elements [22], may be incorporated into composites with other materials [23], and various other reaction parameters (e.g., temperature, solvent, reaction time, precursor ratios, etc.) [24] may all be employed to control optical properties and affinity towards target analytes. As a result, CDs have been widely explored for metal ion detection [25,26,27], a potential boon for the critical minerals sector. Iron in particular is a common target for CD sensors, and multiple comprehensive reviews have been written on this topic [28,29,30]. Fe detection has been achieved from CDs derived from a wide range of both naturally occurring and engineered carbon precursors. Despite the vast literature on carbon dot-based iron sensors, most CD sensors rely on solvothermal or carbonization processes, where carbon precursors are exposed to high temperatures (up to several hundred degrees) for extended periods (ranging from hours to days) and/or require specialized equipment such as microwave or pyrolysis reactors, making simpler, cheaper, and faster approaches desirable [31]. Recently, acid–base reactions have been successfully exploited for CD syntheses. Here, a strong acid reacts with an organic base in the presence of a carbon precursor, creating rapid localized heating to form the CDs; in addition to providing heat to drive the chemical reaction, the choice of acid and base also influences CD functionalization [32,33,34,35,36]. The acid–base mediated approach for CD fabrication offers a potentially scalable, inexpensive, and convenient method for optical sensor development. Adapting CD sensors typically synthesized from tedious solvothermal techniques to an acid–base approach is a straightforward path towards sensor design. As a proof-of-concept, here CDs are synthesized by reacting phosphoric acid and ethanolamine in the presence of m-phenylenediamine for iron sensing; the synthesis was inspired by several literature reports in which CD sensors for iron were prepared using solvothermal approaches [37,38,39,40,41,42].

In this study, the synthesis of iron(III)-responsive CDs was conducted through a bottom-up method of an exothermic acid–base reaction between phosphoric acid and ethanolamine in the presence of m-phenylenediamine. The choice of reagents was inspired by several iron sensors reported in the literature, each of which used m-phenylenediamine as the carbon source (and, by extension, included nitrogen-doping) [38,39,40,41,42]; phosphoric acid was selected as the acid to potentially induce phosphorus doping, an approach used by Dong and co-workers [37]. By adapting these previously reported approaches into an acid–base method, the reaction time is significantly reduced, the need for external heating and high pressure is removed, and expensive starting materials are avoided. The CDs were characterized by X-ray photoelectron spectroscopy (XPS), Fourier-transform infrared spectroscopy (FTIR), transmission electron microscopy, steady-state and time-resolved photoluminescence spectroscopy, and absorption spectroscopy. The CDs exhibited a blue-green emission band that was selectively quenched (relative to other common metals in environmental streams) by iron, with a detection limit in the part-per-billion range even in 0.1 M HCl, highlighting the potential for this material to detect iron in metal processing streams. Importantly, the CDs were also evaluated in realistic samples including Fe-spiked AMD, real AMD solids leachates, and process streams generated from AMD solids leachate passed through an ion exchange column. Analyzing the sensor performance in streams relevant for mining, with low pH and high concentrations of potentially interfering metals, addresses a shortcoming of previous reports on iron sensing which typically have focused on iron detection in biological or freshwater matrices [30].

After benchmarking the sensor performance on commercial instrumentation, a “minimal cost” approach was employed in which the sensing performance of CDs that underwent minimal purification was evaluated using a lab-built portable fiber optic luminescent spectrometer [43,44], focusing specifically on the materials’ sensitivity, selectivity, and stability under environmentally relevant conditions, with low parts-per-million sensitivities in acidic, high ionic strength matrices. The compact fiber optic spectrometer platform is particularly convenient for real-time process monitoring or even metal prospecting due to its low power requirement and size. A “test strip” approach is also demonstrated, in which CD-coated filter paper is used with a compact analyzer for iron detection, providing yet another convenient approach for iron ion characterization. Overall, this work demonstrates the rapid synthesis of CDs which, when coupled with an inexpensive and compact fiber optic spectroscopic probe, could aid in the quantification of iron(III) through real-time on-site measurements, yielding a beneficial approach for the recovery of critical minerals from domestic sources.

## 2. Materials and Methods

Materials. o-phosphoric acid (85 wt. % in $H_{2}O$), pH 1.68 buffer (potassium tetroxolate dihydrate, ${Orion}^{TM}$), and potassium chloride (certified ACS) were purchased from Thermo Fisher Scientific (Waltham, MA, USA). Cobalt(II) nitrate hexahydrate (99+%), aluminum(III) nitrate nonahydrate (99+%), and iron(III) nitrate nonahydrate (99+%) were purchased from Acros Organics (Geel, Belgium). Neodymium(III) nitrate hexahydrate (99.9%), iron(II) sulfate heptahydrate (ACS reagent, 99.0%), molecular sieves (4 Å, powder), sodium chloride (ACS reagent grade), calcium chloride (anhydrous), copper(II) nitrate trihydrate (99–104%), zinc nitrate hexahydrate (99.999%), manganese(II) nitrate tetrahydrate 97.0%), reagent grade m-phenylenediamine (99%) and ethanolamine (≥98%) were purchased from Sigma Aldrich (St. Louis, MO, USA). Magnesium nitrate hexahydrate (ACS, 99.8–102.0%) and nickel(II) nitrate hexahydrate (98%) were purchased from Alfa Aesar (Haverhill, MA, USA). De-ionized water (purity of 18.2 MΩ·cm, Barnstead EASYpure LF system (Thermo Fisher Scientific, Waltham, MA, USA)) was used to prepare all aqueous solutions.

Synthesis of CD Sensing Material. CDs were synthesized using an acid–base reaction by weighing approximately 0.05 g of m-phenylenediamine to which 0.5 mL of ethanolamine (≥98% grade) was added in a glass vial. The mixture was vortexed and sonicated until the m-phenylenediamine dissolved completely. Under a fume hood 166 μL of concentrated (85% by weight) phosphoric acid were rapidly added to the glass vial to produce a 1:1 acid–base ratio. The resulting solution was gently swirled and an exothermic reaction proceeded, resulting in a white and brown solid. The reagents were used at room temperature without any external heating; heat generated by the acid–base reaction drove the CD formation. Monitoring the reaction with a thermocouple meter (Digi-Sense Data Logging Thermocouple Meter, 20250-92, manufactured by Cole Parmer, Vernon Hills, IL, USA) revealed the reaction reached a temperature of 110 °C 45 s after phosphoric acid addition and gradually cooled to 26 °C after 10 min. After 10 min, 5 mL of methanol was added while sonicating and stirring with a metal spatula to selectively dissolve the CDs, followed by centrifugation at 8000 rpm for 5 min; the supernatant was saved while all precipitates were discarded. This centrifugation step was repeated two additional times, followed by purification through an alumina column using methanol as the mobile phase. Finally, the CDs were filtered through a 0.22 μm PTFE syringe filter and stored at 4 °C.

“Minimal Purification” CD Synthesis. Because lengthy and tedious purification processes are one of the more time-intensive and costly steps for CD synthesis, a minimal purification approach was used and evaluated to minimize costs for iron detection. These CDs were synthesized following the same approach described above, but following cooling after phosphoric acid addition, 3 mL of deionized water was added instead of methanol, followed by centrifugation.

Photoluminescence Characterization using a Commercial System. The optical properties and sensing performance of the purified CDs were benchmarked on a Horiba Jobin-Yvon Fluorolog 3 (Horiba, Kyoto, Japan) equipped with FluorEssence software (V3.1.5.11) and a 450 W xenon lamp. Excitation and emission slits were set to 5 nm, with a 0.1 s integration time. A 380 nm cut-off filter (Edmund Optics, Barrington, NJ, USA) was used to block excitation light from reaching the detector. Quartz cuvettes (Thorlabs, Inc., Newton, NJ, USA) were used for all measurements. In a typical experiment, a solution with an optical density of 0.2 (at 365 nm, measured by UV-Vis) was prepared by adding filtered CDs to 3 mL of a sample matrix. For limit of detection studies, 0.1 M Fe(NO_3_)_3_ was titrated into the CD solution, and the Fluorolog was programmed to measure intensity at the emission maximum (500 nm) every 0.1 s for 10 s, producing 100 data points for each concentration level of iron tested. For selectivity tests, the emission spectrum of 3 mL of 0.2 optical density (at 365 nm) CDs was measured before and after the addition of 15 μL of 0.1 M solutions of different potentially interfering metals; the spectrum was then recorded again following the addition of 15 μL of 0.1 M iron(III) nitrate.

Luminescent Measurements Using a Portable Fiber Optic Spectrometer. The sensing performance of the “minimal purification” CDs was evaluated on a lab-built portable fiber optic spectrometer described previously. A description and photograph of the system may be found elsewhere [45], along with a block diagram [16]. During the LOD experiment, the response of the sensor was evaluated as a function iron(III) concentration. This was done by adding 10 mL of water and 250 μL of the CD stock solution to a glass vial and pipetting in increasing amounts of iron, as described above. A 3 s integration time was used for all measurements, with 3 scans averaged, and a “dark” spectrum was collected and subtracted for all spectra. The strip chart function on the QEPro (Ocean Optics, Orlando, FL, USA) was used to monitor the peak area from 420 nm to 680 nm at each iron concentration, collecting at least 7 data points at each concentration level. All studies were conducted while stirring at 850 rpm using a stir plate and magnetic stir bar.

Time-resolved Photoluminescence. Time-resolved photoluminescence studies were conducted on CDs (~0.2 OD at 365 nm) in solution using a Picoquant FluoTime 300 (Picoquant, Berlin, Germany) spectrometer equipped with a 375 nm pulsed laser (LDH-PC-375) excitation source and a time-correlated single photon counting detector (TimeHarp260P, Picoquant, Berlin, Germany). Emission counts were monitored at 500 nm and were collected until 10,000 peak counts were obtained. A 450 nm cut-off filter (Thorlabs Inc., Newton, NJ, USA) was used. Iron(III) nitrate was titrated in and measurements were collected after each addition.

Limit of Detection Calculation. A calibration curve was generated by plotting the Stern-Volmer relationship:

(1) $$\left(\frac{I_{o}}{I}-1\right)=m[Q]$$

Here “*I*” represents the peak intensity/area following an addition of iron, *I_o_* denotes the peak intensity/area measured of the CDs alone (with no iron added), and *Q* represents the concentration of iron added. The slope “*m*” of the plot was utilized to estimate sensitivity. To approximate the noise, the average intensity of the sample without iron ($I_{o})$ was divided by the intensity of each data point collected at the lowest iron concentration analyzed. The standard deviation (*s*) of these values was then calculated, and the limit of detection was determined using the obtained standard deviation using the formula:

(2) $$LoD=3\;\times \frac{s}{m}$$

The limit of quantification was determined using the formula:

(3) $$LoQ=10\;\times \frac{s}{m}$$

Selectivity Tests Using Portable Spectrometer. The selectivity of the sensor against other metals commonly encountered in coal streams was evaluated by adding 10 mL of water and 250 μL of the CD stock solution to a glass vial then adding an individual metal. The luminescent properties of the CDs were measured before and after adding 50 µL of 1 M solution of each metal tested and then the luminescent properties were recorded once again after 50 µL of 1 M iron(III) was added. In between testing each interfering metal, the glass vial was rinsed three times with D.I water to ensure there was no contamination.

Monitoring of Acid Mine Drainage Solid Leachates in an Ion Exchange Column Process. A strong acid cation ion exchange resin was employed in a glass column for selective uptake of rare earth elements from a leachate solution. This leachate was obtained from the hydrochloric acid leaching of AMD solids. The objective of this process was to separate rare earths from other co-existing ions and to quantify the selectivity of the chosen resin for rare earths. For this purpose, 572 g of a commercially available ion exchange resin, DuPont Amberlite HPR1200-H (Dupont, Wilmington, DE, USA), was packed into the glass column.

The column featured 270 mL of pore space between the resin beads. An average flow rate of 2.5 L/h was maintained for the flowing leachate, resulting in an average residence time of 6.5 min within the packed column. Samples were collected approximately every 3 L to monitor pH, density, and electrical conductivity. The complete ionic composition of the samples was determined using Inductively Coupled Plasma-Optical Emission Spectrometry (ICP-OES) for major ions and ICP-MS for minor ion species. The samples were analyzed using a commercial system as described above. Similarly, AMD and AMD solids leachates in formic acid and acetic acid were obtained, characterized by ICP-MS, and were analyzed at various dilution levels in the presence of CDs.

Ultraviolet-Visible (UV-Vis) Absorption Spectroscopy. UV-Vis spectroscopy measurements were taken using a PerkinElmer Lambda 1050 (PerkinElmer, Waltham, MA, USA) spectrophotometer by adding CD stock solution to 2 mL of water.

X-ray photoelectron spectroscopy (XPS). XPS spectra of CDs with and without iron exposure were acquired with a ULVAC-PHI Versa Probe III instrument (Physical Electronics, Chanhassen, MN, USA) using a focused (100 μm) monochromatized Al Kα X-ray source (1486.6 eV) and dual charge neutralization. The electron and ion neutralization current densities were set at 22 nA/mm^2^ and 250 pA/mm^2^, respectively. A pass energy of 55.0 eV was used for all high-resolution scans, whereas a pass energy of 140 eV was used for the collection of survey spectra. High-resolution spectra were charge-corrected using C 1s = 284.8 eV as a reference energy. Samples were prepared by dropcasting 200 μL (in 50 μL increments) of CDs onto a glass slide. For the iron-exposed sample, 5 μL of 1 M iron(III) chloride was added to each 50 μL CD aliquot.

Transmission Electron Microscopy (TEM). TEM characterization was performed on a Hitachi H9500 Environmental TEM (Hitachi, Ibaraki, Japan) with an accelerating voltage of 300 kV (NanoScale Fabrication and Characterization Facility, Petersen Institute of NanoScience and Engineering, Pittsburgh, PA, USA), equipped with a Gatan Orius camera (Gatan, Warrendale, PA, USA). The stock solution of the purified CDs was diluted 1:25 in water and was dropcast onto an ultrathin carbon-coated holey TEM grid (Ted Pella, Inc., Redding, CA, USA).

X-ray Diffraction. XRD pattern was collected using a Malvern Panalytical Empyrean X-ray diffractometer (Malvern, Worcestershire, UK) with Cu radiation (kα = 0.1541877 nm) operated at 45 kV and 40 mA. The sample was placed on a zero-background silicon holder (200 μm depth) and was scanned at the spinning stage with a step size of 0.01313 degrees. The scan time was 1000 s/step (scan rate: 0.003350 deg/s) with a total analysis time of 6 h and 5 min.

Fourier-Transform Infrared Spectroscopy. Reagents and samples were analyzed using Attenuated Total Reflectance-Fourier Transform Infrared Spectroscopy (ATR-FTIR) to compare the major functional groups on the CDs and CD reactants. A Vertex 70 (Bruker, Billerica, MA, USA) Fourier transform infrared (FT-IR) spectrophotometer equipped with a GladiATR (Pike Technologies, Madison, WI, USA) diamond ATR accessory was utilized to measure the infrared spectra of the samples.

Fabrication of iron-responsive test strips. Iron sensing test strips were fabricated by pipetting 1 mL of dilute CDs (prepared by mixing 333 μL of the “minimal purification” CD stock solution with 667 μL D.I.water) onto 90 mm diameter ashless round filter paper substrates, wetting the entire paper, and drying in a lab oven set to 80 °C for 7 min. The large filter paper was then cut into smaller pieces and exposed to metal solutions and varying concentrations of iron(III) independently. Images were recorded by an Apple iPhone XS Max under room lighting and under a handheld (Analytik Jena, Thuringia, Germany, model UVGL-55 6 W) 365 nm UV lamp illumination. Quantitative measurements were made using a test strip analyzer (Quick Strip model) from Fluid Photonics (Plainview, NY, USA), using the 365 nm LED source. The instrument records intensity at 500 nm. Each test strip was exposed to different concentrations of iron(III), and 7 replicates were conducted at each concentration tested. Limits of detection were estimated using the procedure described above. Stability tests over the course of two weeks were conducted in triplicate on test strips stored in air, in a parafilm-covered beaker containing molecular sieves, and in a refrigerator set to 4 °C; emission intensity of the films was monitored using the Quick Strip analyzer.

## 3. Results and Discussion

### 3.1. Characterization of Purified CDs

**Figure 1:**
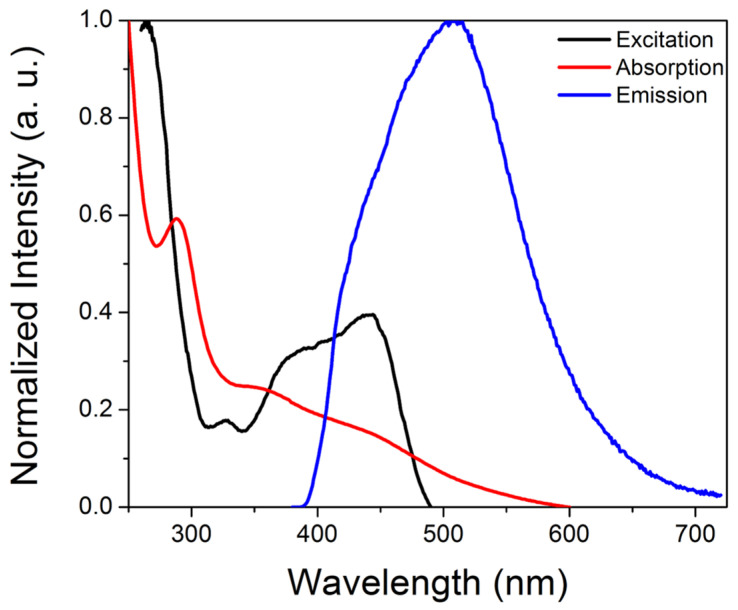
Representative absorption (red), excitation (black) and emission (blue) spectra of the iron-responsive CDs.

**Figure 2:**
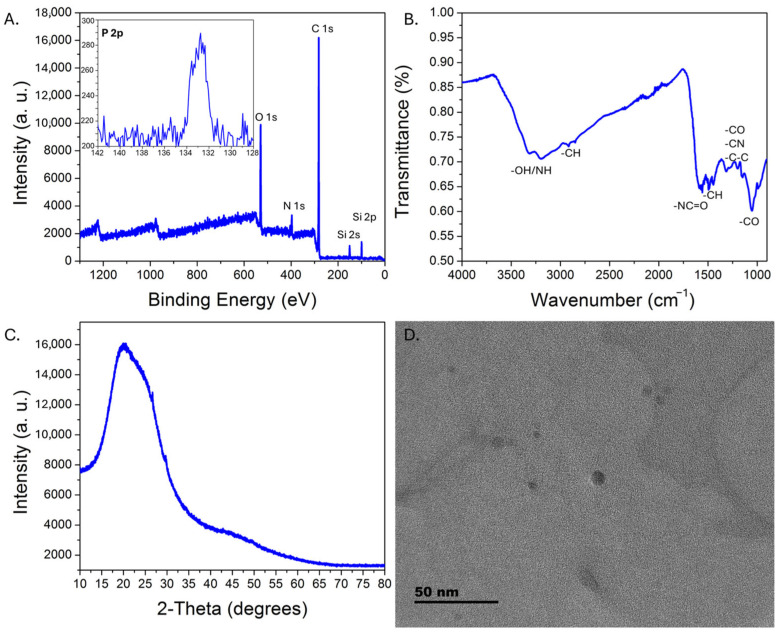
Physical characterization of the CDs. XPS survey spectrum of the CDs (**A**), with an inset showing the high-resolution weak P 2p spectrum. Si signal is from the glass substrate. FTIR-ATR transmission spectrum (**B**) illustrates the major functional groups on the CDs. The XRD pattern for the CDs is shown in (**C**). A representative HRTEM image (**D**) demonstrates pseudospherical morphology with sub-10 nm diameters.

Upon UV excitation, the purified CDs exhibited green emission centered at ~510 nm, consistent with observations reported for solvothermally synthesized m-phenylenediamine-derived CDs, with comparable absorption (~290 nm, 365–450 nm broad peak) and excitation (265 nm, 365–450 nm broad peak) features (Figure 1) [37,38,40,46]. A 365 nm excitation wavelength was selected because (a) higher excitation wavelengths would overlap with the emission peak, which would prevent monitoring of the whole emission peak during sensing experiments, and (b) 365 nm excitation sources for portable instrumentation are common, enabling better comparisons between studies on commercial systems versus portable set-ups. For applications in which only the emission maximum is monitored, improved sensitivity may potentially be realized by exciting at higher wavelengths (e.g., 450 nm). The quantum yield, estimated relative to quinine sulfate standards, was 9.6 ± 0.8%, which is lower than values reported for the solvothermally synthesized m-phenylenediamine carbon dots [37,38,39]. XPS analysis revealed significant nitrogen doping but minimal phosphorus incorporation, despite the use of phosphoric acid; the composition determined by XPS was 79.6% C, 13.2% O, 7.1% N, and 0.1% P (Figure 2A). The P that was incorporated into the carbon dots has a binding energy of ~132.8 eV, consistent with phosphate groups. FTIR-ATR analysis (Figure 2B) indicated amine/hydroxy functional groups (~3180, 3310 cm^−1^), C-O (~1050 cm^−1^), amide groups (~1550–1580 cm^−1^ and ~1312 cm^−1^), with C-H bending (~1450 cm^−1^) and C-O/C-N/C-C stretching (~1195 cm^−1^ and 1145 cm^−1^) also observed. XRD characterization revealed a broad peak centered near 20 degrees 2-theta, consistent with CD formation [47,48] (Figure 2C), while HRTEM analysis (Figure 2D) indicated a pseudospherical morphology with an average diameter of ~6 ± 2 nm (Figure S1).

### 3.2. Sensing Performance Evaluation on Commercial Spectrometer

**Figure 3:**
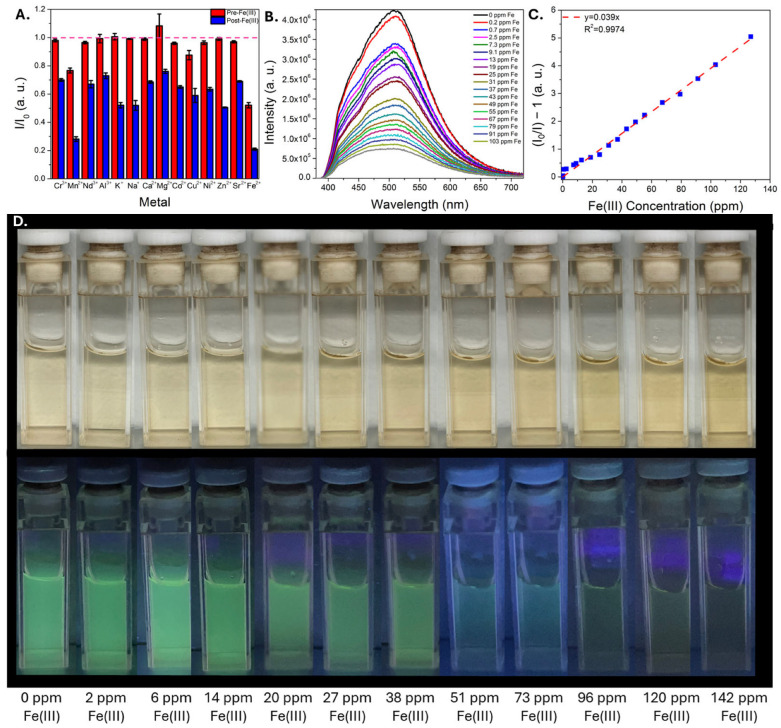
Plot of CD intensity change after adding a potentially interfering metal (red bar) followed by the addition of iron(III) (blue bar) (**A**). A dotted line is included for comparison at an intensity ratio equal to 1, in which no quenching is observed. CD emission spectra in water as a function of increasing iron(III) concentration (**B**), with the corresponding Stern-Volmer plot illustrating linear quenching (**C**). Photographs of CDs exposed to increasing iron(III) concentration are shown in (**D**) under ambient and 365 nm light.

The CDs were then screened for their ability to detect metal ions by monitoring their optical properties in the presence of some of the most common metals in coal utilization byproduct streams. Among the metals tested, iron ions induced the most significant change, selectively quenching CD emission when introduced (Video S1), which is consistent with previous reports on m-phenylenediamine-derived CDs synthesized using solvothermal methods [37,40]. Selectivity studies were conducted (in triplicate) in which changes to CD emission properties were measured before and after exposure to (a) a potentially interfering metal, then (b) iron added to the same solution. These results indicated that the CDs exhibit good selectivity for iron without significant interference from other common metals in coal utilization byproduct streams (Figure 3A). Although both iron(II) and iron(III) induced appreciable quenching, sensing metrics were benchmarked using iron(III) due to its prevalence in coal utilization byproduct streams [49]. Sensitivity tests were then conducted in which the quenching of CD emission was monitored as a function of iron concentration; these experiments were conducted in both deionized water (Figure 3B–D) as well as 0.1 M HCl (Figure S2) to determine how well the sensor can function in acidic environments that would likely be encountered in metal processing streams. Remarkably, the limit of detection values in both matrices were very similar: 0.5 ± 0.1 ppm was estimated in both conditions, implying that acidic conditions do not significantly hinder the ability of the CDs to detect iron. Taken together, these results indicate that the Fe-responsive m-phenylenediamine solvothermal reaction is adaptable to an acid–base mediated synthesis, a promising step for improving scalability and reducing the time and financial costs associated with material synthesis.

**Figure 4:**
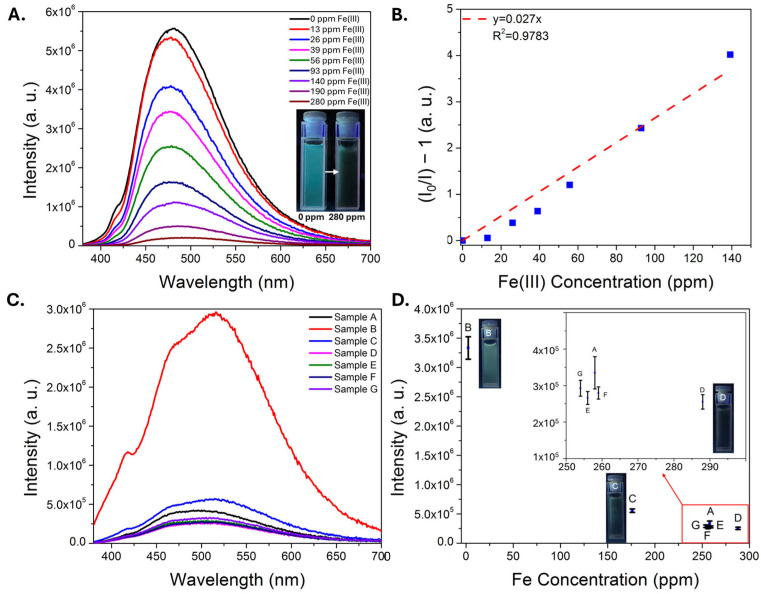
Plot of CD intensity in an AMD sample with increasing iron(III) nitrate spikes (**A**). The inset is a photograph of CDs in unspiked AMD and with a 280 ppm Fe(III) spike under illumination with a 365 nm lamp. A Stern-Volmer plot (**B**) demonstrates a linear response up to ~150 ppm in this matrix. Plot of CD emission spectra (**C**) in different ion exchange column fractions collected from a real AMD solids leachate (Sample A is the raw sample). A plot of intensity versus ICP-MS determined iron concentration (**D**) indicates good correlation between emission intensity and iron concentration, with photographs of several samples under UV illumination and an inset plot to zoom in on similar data points. Error bars represent the standard error of three independent trials. The letters A–G are used to match the individual points in Plot D with the spectra shown in Plot C.

After evaluating the sensing performance of the CDs in acid and in the presence of other metals, the carbon dots were then deployed into a real sample of AMD to confirm whether the sensor could function in such a matrix. The AMD sample naturally had low (0.3 ppm) concentrations of iron (Table S1), with a pH of 3 and high concentrations of aluminum (7.5 ppm), silicon (10.8 ppm), calcium (67 ppm), magnesium (47 ppm) and manganese (14 ppm). Iron(III) nitrate was spiked into the AMD and the CD emission was recorded in this matrix. Importantly, strong CD emission was observed in the unspiked AMD, and a linear response to iron(III) was observed up to ~150 ppm (Figure 4A,B). Reported iron concentrations can vary significantly in AMD, from negligible (part-per-billion levels) to over 1000 parts-per-million [14]. These results suggest that the sensor could thus have utility for tracking iron removal in an AMD treatment site as one potential application area [50,51]. To evaluate sensor performance in a matrix with higher concentrations of potentially competing ions, a similar study was conducted on an AMD solids leachate in sulfuric acid, diluted by a factor of 20 so that the naturally occurring Fe concentration was at the lower end of the linear dynamic range (~15 ppm). The pH of the diluted sample was ~2.7 and the sample contained 510 ppm Al, 230 ppm Si, 30 ppm Ca, and 20 ppm Zn, among other common metals (Table S1). A consistent reduction in emission signal was observed as a function of added iron concentration, with reductions observed even at sub-ppm Fe additions (Figure S3). Encouraged by these results, the CDs were then used to monitor a process in which a real AMD solids leachate was run through an ion exchange column. CDs were added to different fractions collected during this process, and the pH and major elemental compositions (as determined by ICP-MS) are included in Table S2. Iron was initially absorbed by the ion exchange resin, and strong emission signal was observed in the sample B, which had low iron content (2 ppm). The iron concentration subsequently increased significantly over the next several fractions, and a corresponding decrease in the emission signal was observed in the samples containing higher iron levels, samples C through G (Figure 4C,D). Notably the iron concentration was outside the expected linear dynamic range of the sensor, however good correlation between emission signal and iron concentration was observed in the samples, suggesting potential utility for the sensing material in process monitoring applications. Finally, to better understand the sensor performance (and limitations) in more challenging samples, the emission properties of the CDs were measured in two real AMD solids leachates at different dilution factors; one sample was an acetic acid leachate while the other was a formic acid leachate, with high concentrations of iron and other metals (Table S3). Very weak emission was observed in the undiluted leachates, likely due to multiple factors including high iron content (>1000 ppm Fe in both cases), high concentrations of non-target metals that may also induce quenching, and the opacity of the samples, which were both colored and cloudy. The CD emission could easily be observed at higher dilution levels where the iron content began to approach the linear dynamic range of the sensor; moreover, the sample with lower iron concentration exhibited stronger emission at these higher dilution levels, suggesting that the sensor may be applied to compare iron content in samples with similar matrices (Figure S4).

Table S4 compares the sensing performance, synthesis properties, and quantum yield of the CDs reported in this work versus other reported iron-responsive m-phenylenediamine CDs. In addition to the ease of synthesis (e.g., shorter reaction time and lower reaction temperature), other potential advantages of the acid–base synthesized CDs include a wide linear dynamic range and the ability to function in highly acidic environments. However, the quantum yield is lower than that of the solvothermally synthesized CDs, with a lower sensitivity compared to several other reported sensors. These differences may be rooted in alterations of surface functionality (e.g., differences in the number, type, and arrangement of surface functional groups as a function of the synthetic approach [52]) which would be expected to impact selectivity as well as synthesis-dependent differences in optical properties (for example, synthesis-dependent differences in the quantum yield).

### 3.3. Low-Cost Sensor Approach

With physical characterization in hand and sensing performance benchmarked against a commercial spectrometer, experimental efforts focused on reducing the cost of the sensing material while integrating it with portable, inexpensive instrumentation for potential deployment in remote areas, such as AMD sites. Using this approach, CDs and reaction byproducts were dissolved in water following the acid–base synthesis and used without further purification. While CD purification is essential to understanding their properties and maximizing their performance [53], purification approaches can be the most costly and time-consuming step in their fabrication; moreover, in harsh environments where refrigeration for CD powders and solutions may not be practical or possible, on-site CD generation may be required without access to centrifuges, dialysis systems, or other purification equipment. In these experiments, the unpurified CDs were paired with a lab-built portable fiber optic luminescence spectrometer platform that we have previously described [16]. The platform utilizes a solarization-resistant bifurcated optical fiber cable that links a 365 nm light-emitting diode (LED) excitation source, the CD sample in solution, and a commercial compact spectrometer. The device is sufficiently compact to fit into a box or backpack for transport into the field [45] with power requirements that could be met using solar panels or batteries [44]. Additionally, because optical fibers can transmit signals across long distances, there is potential to use such a platform to access environments that may be difficult to reach; here, a 2 m fiber cable is used; however, the setup could also be integrated with longer fiber sections as needed.

**Figure 5:**
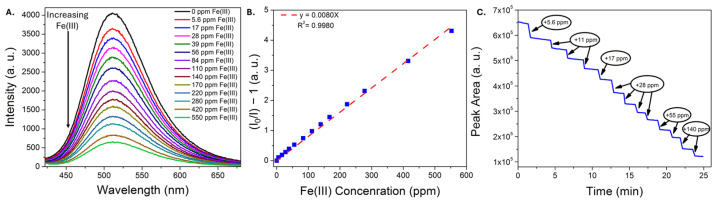
Representative emission spectra (**A**) of CDs in water exposed to increasing concentrations of iron(III) nitrate, recorded using a portable fiber optic sensor. (**B**) The corresponding Stern-Vomer plot used to estimate the limit of detection for iron(III). (**C**) Plot of emission peak area as a function of time, where different amounts of iron(III) are added. An immediate drop in emission intensity is noted upon each iron(III) addition.

Representative data illustrating the response of the CDs to Fe(III) ions using the portable platform are shown in Figure 5. The characteristic green emission band of the CDs is quenched with increasing iron concentration (Figure 5A), exhibits a linear Stern-Volmer profile up to at least 600 ppm Fe(III) (Figure 5B), and, when monitoring the peak area over time, the emission responds almost instantly upon Fe(III) addition. The linear dynamic range achieved using the portable set-up was longer than that achieved using a commercial instrument; we have observed this previously using a carbon dot sensor [16] and attribute this in part to the use of a weaker excitation source, a factor that has been reported to increase the linear dynamic range [54]. Other factors may include different excitation and emission collection paths in the portable set-up [55], and/or the use of less pure sensing material. A limit of detection of 2.2 ± 0.3 ppm Fe(III) was determined from three independent trials in water. Subsequent analyses of the limit of detection of Fe(III) in a pH 1.68 buffer (Figure S5), representing an acidic, high-ionic-strength matrix, yielded a similar detection limit of 2.83 ± 0.06 ppm, demonstrating that the sensing material remains functional in acidic matrices even with minimal purification.

**Figure 6:**
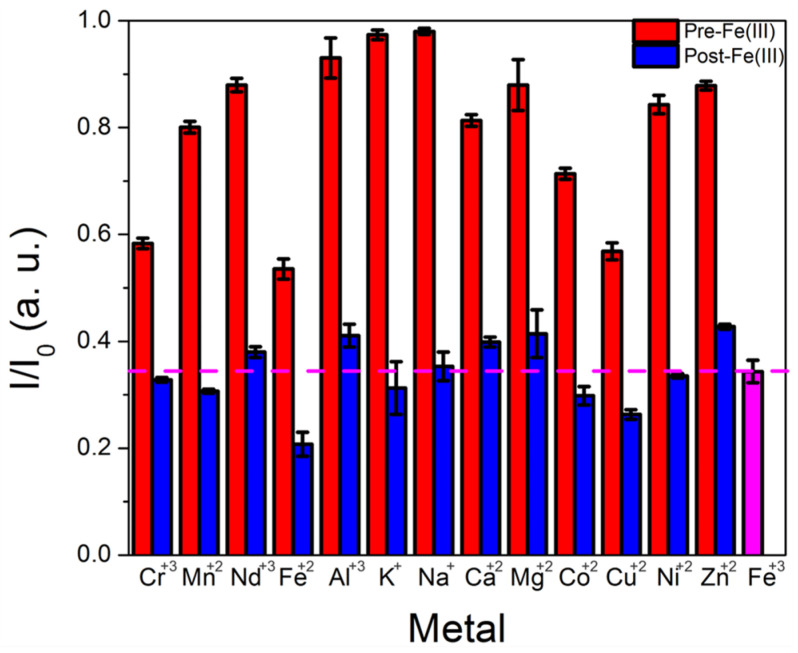
Plot of CD intensity change after adding a potentially interfering metal (red bar) followed by the addition of iron(III) (blue bar), measured using the portable fiber optic spectrometer with CDs that underwent minimal purification. A dotted line is included at the quenching levels observed when iron(III) alone is added (signified by the pink bar) to compare the effects of iron(III) alone versus iron(III) added in the presence of other metals.

Selectivity measurements in which the CD emission was monitored before and after addition of a potentially interfering metal, followed by a second measurement in which Fe(III) was added, indicated a slight decrease in performance compared with results obtained using purified CDs and a commercial system; Cu(II) and Cr(III) also quenched CD emission by over 20%, although iron(III) and iron(II) induced significantly more quenching (~35%). Moreover, the presence of the additional metals did not substantially interfere with iron’s ability to quench CD emission (Figure 6). Any cross-sensitivity to other metals can reduce a sensor’s utility in certain applications, particularly those where the target metal concentration is comparable to or lower than that of competing metals. However, in the case of coal utilization byproduct streams, iron is typically present at orders of magnitude higher concentrations than copper or chromium, and cross-sensitivity to these metals is less of a barrier for practical deployment as a result.

### 3.4. CD Test Strips for Iron Analysis

**Figure 7:**
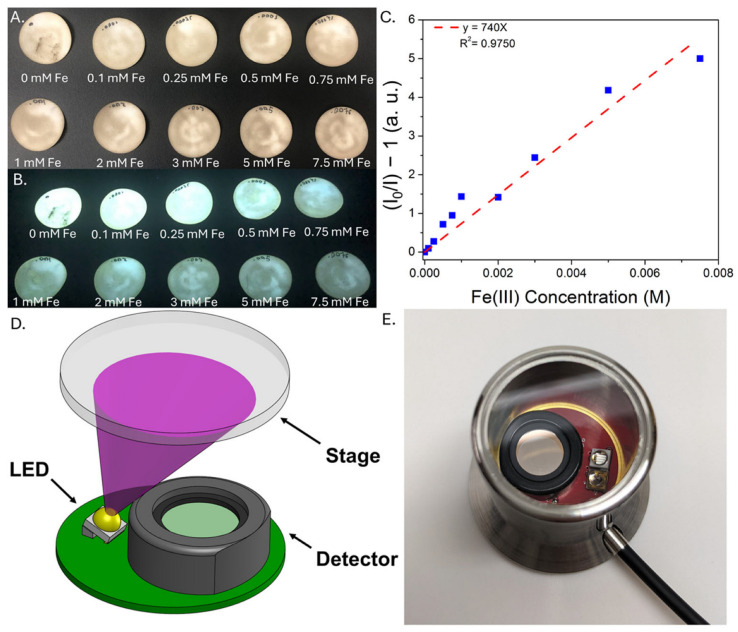
CD-coated filter paper exposed to increasing concentrations of iron(III) nitrate in water under ambient (**A**) and 365 nm (**B**) lighting, indicating quenching of the emission as a function of iron concentration. (**C**) corresponding Stern-Volmer plot of quenching recorded on the test strips using a commercially available film analyzer. A schematic of the Fluid Photonics Quick Strip analyzer is shown in (**D**), along with a photograph of the device (**E**).

To further enhance the portability and versatility of the CD sensors, a separate approach was developed in which the CDs were dried onto sections of filter paper, which were used as visual test strips for iron analysis. Analogous to pH paper, the CD-coated filter paper may be used as a quick and convenient indicator to gain qualitative insight into iron concentration, an important consideration for monitoring iron removal, for example, or characterizing iron content in an AMD site. Similar to the CDs dispersed in water, the emission from the CD-coated filter paper is quenched upon exposure to increasing iron concentration (Figure S6), and this quenching is much more pronounced for iron than any of the metals most commonly found in coal utilization byproduct streams (Figure S7). A more quantitative analysis using the iron test strips is shown in Figure 7. Here, circular sections of CD-coated filter paper were prepared and exposed to various concentrations of iron(III); emission at 500 nm was then analyzed in triplicate. Representative images of test strips under ambient and UV light are shown in Figure 7A, as well as a representative calibration curve used for the iron(III) analysis (Figure 7B). Figure 7C,D illustrate the basic set-up of the Fluid Photonics Quick Strip analyzer, which consists of a 365 nm LED, a quartz imaging stage/sample holder, and a photodiode detector beneath a 500 nm bandpass filter. A block diagram of the device is included in Figure S8. Despite inevitable batch-to-batch variations in the emission properties of individual filter paper sections, a linear correlation was observed between iron content and emission, with a detection limit of ~8 ± 1 ppm Fe estimated across three independent trials. This provides a simple method for quantitatively analyzing test strip emission for rapid, portable iron analysis. Because the device records only at the emission maximum, we note that shifts in the emission wavelength due to matrix effects could complicate interpretation of the sensor response; however, this could be mitigated by, for example, using a bandpass filter with a wider range, as the test strip reader may be customized for specific experimental needs. While these results are a promising step towards portable sensor deployment, stability tests conducted over the course of two weeks indicate that the emission properties of test strips stored in air degrade rapidly over the course of several days; although storage techniques such as refrigeration and/or desiccation enhance the stability of the test strips, on-site preparation of test strips followed by same-day testing is currently the most practical approach (Figure S9).

The level of material processing and platform sophistication may thus be balanced against experimental requirements. When high precision is required in a controlled laboratory environment, column-purified CDs coupled with a commercial spectrometer provide the highest levels of sensitivity (0.5 ppm). However, in remote areas where access to a commercial system is not possible, the portable fiber optic spectrometer with minimally purified CDs may achieve detection limits down to ~2.2 ppm while providing the entire emission spectra. In circumstances where a more qualitative “spot check” is sufficient, the most convenient option would be the test strip reader, with an 8 ppm limit of detection. One can make an analogy to pH analysis, where in many cases pH paper is the most convenient option to monitor pH, but when greater precision is needed a pH probe is used. Here, one can envision using the test strip to estimate Fe content, followed by more precise measurements as needed using either a portable device or a commercial spectrometer.

### 3.5. Mechanism for Iron Ion Detection

Additional insights into the sensing mechanism were explored through absorption spectroscopy and time-resolved luminescence spectroscopy, where measurements were first made on CDs alone, followed by the addition of iron(III). UV-Vis analysis (Figure S10) reveals a significant increase in absorbance when Fe(III) is added to a CD solution; this increase in absorbance is substantially higher in magnitude than the sum of the individual CD and iron spectra and is consistent with the formation of iron complexes with CD functional groups [56]. Moreover, the peak shape of the CD-iron sample is significantly narrower than that of iron(III) alone. Luminescent lifetime measurements indicate an average lifetime of 7.4 ± 0.2 ns for the CDs (three independent trials), similar to other reports of m-phenylenediamine CDs [39,40]. Upon the addition of iron(III), a slight decrease in lifetime is observed at higher (e.g., >100 ppm Fe) concentrations, and the magnitude of this lifetime decrease correlates with added iron (Figure S11). The formation of a complex between the CDs and iron, as indicated by UV-Vis, suggests a static quenching mechanism. XPS analysis of the CDs before and after iron exposure indicates both oxygen and nitrogen-containing functional groups on the CD surface are involved in iron chelation, providing further evidence of complex formation as evidenced by binding energy shifts and new peaks in the C 1s, N 1s, and O 1s spectra (Figure S12A–C). Fe 2p signal was also observed upon chelation (Figure S12D). The low (~0.1%) incorporation of P suggests that phosphorus-containing species are unlikely to play a significant role in the sensing mechanism; moreover, several iron-responsive m-phenylenediamine CDs have been reported that do not contain P-doping [38,39,40,41,42], suggesting this is not necessary for the sensor response. The decrease in the luminescent lifetime upon exposure to increasing iron content also indicates that dynamic quenching processes (e.g., energy transfer and/or collisional quenching) are occurring; however, this decrease in lifetime is only obvious at high (>100 ppm) iron concentrations, suggesting static quenching is the primary mechanism. Notably, there is partial overlap between the iron(III) absorption spectrum and the CDs excitation spectrum (Figure S13), which indicates that inner filter effects also likely contribute to the quenching mechanism. Additional evidence of multiple quenching pathways at higher iron concentrations is also evident in the upward-curving Stern-Volmer plot at iron concentrations beyond the linear dynamic range (Figure S14) [57]. The combination of both quenching mechanisms has been reported previously for iron-responsive m-phenylenediamine CDs [39,40]. A definitive determination of the source of selectivity towards Fe(II)/Fe(III) versus other metal ions is more difficult to experimentally establish; however the surface functional groups on the CD surface presumably play a critical role in modulating selectivity. Hard-soft acid base theory is one mechanism by which CD selectivity is thought to be governed, where nitrogen and oxygen-rich functional groups are predicted to have a high affinity for iron; there are multiple examples of this design rule being successfully employed in the design of iron-responsive CDs [25]. Moreover, other reports of iron-responsive m-phenylenediamine CDs have attributed selective chelation by surface functional groups as the chief cause of iron specificity versus other similar metals [38,39,41].

## 4. Conclusions

Here, we demonstrated the adaptation of a solvothermal synthesis of an iron-responsive CD into an acid–base approach, offering potential savings in terms of time, equipment cost, and energy consumption. Purified CDs were physically characterized, and their sensing performance for iron(III) was benchmarked on a commercial spectrometer, demonstrating sub-parts-per million limits of detection in both water and acidic environments as well as selectivity against most metals commonly encountered in environmental streams. For applications in remote areas, such as acid mine drainage sites, a minimal cost approach was conducted in which the CDs were not subjected to purification and were evaluated using an inexpensive and portable fiber optic probe. Moreover, CD-coated filter paper strips were also evaluated as a second sensor modality for iron detection, and their performance in detecting down to ~8 parts per million of Fe(III) in water was demonstrated using an inexpensive and compact analyzer. While a plethora of CD materials have been reported for iron detection in solution [28,29], areas of novelty in this work include (a) the design of a CD probe for iron using an acid–base synthesis approach, (b) integration of the iron-responsive CDs with portable optical probes, and (c) the focus on iron detection specifically for coal utilization byproduct streams, which are of increasing importance due to their use as a source of economically critical metals.

## Data Availability

The data presented in this study are not publicly available at this time but may be obtained upon reasonable request from the authors.

## Supplementary Materials

The following supporting information can be downloaded at: https://www.mdpi.com/article/10.3390/s25196066/s1, Video S1. Emission quenching of CDs by Fe(III) under UV illumination; Figure S1. Size distribution histogram of the carbon dots as determined by high-resolution transmission electron microscopy and additional TEM images; Figure S2. Representative emission spectra and Stern-Volmer plot for carbon dots exposed to increasing concentrations of iron(III) in 0.1 M HCl, measured using a commercial spectrometer; Table S1. ICP-MS characterization of the AMD and AMD solids leachate in sulfuric acid (after 1:10 dilution) samples; Figure S3. Emission spectrum and plot of maximum intensity versus added Fe(III) concentration of the carbon dots in a sulfuric acid AMD solids leachate; Table S2. pH and ICP-MS characterization of ion exchange column fractions of AMD solids leachate; Table S3. ICP-MS characterization of acetic acid and formic acid leachates from AMD solids; Figure S4. Emission spectra of CDs in a formic acid AMD solids leachate and in an acetic acid AMD solids leachate at different levels of dilution; Table S4. Comparison of optical properties, synthesis, and sensing performance of iron-responsive m-phenylenediamine-derived carbon dots; Figure S5. Representative emission spectra and Stern-Volmer plot for carbon dots exposed to increasing concentrations of iron(III) in pH 1.68 buffer, measured using the portable fiber optic spectrometer; Figure S6. Photographs of carbon dot-coated filter paper exposed to increasing concentrations of iron(III) nitrate; Figure S7. Photographs of carbon dot-coated filter paper exposed to 1M aqueous solutions of different metals commonly found in AMD and other coal utilization byproducts under ambient and 365 nm light; Figure S8. Block diagram of the Quick Strip analyzer for quantitative detection of iron using carbon dot-coated strips and a photograph of the analyzer in action; Figure S9. Analysis of test strip emission recorded using the Fluid Photonics Quick Strip analyzer over the course of 2 weeks under different storage conditions; Figure S10. Absorption spectra of carbon dots in water before and after the addition of 19 and 37 ppm iron(III) nitrate; Figure S11. Time resolved luminescent decays of carbon dots before and after exposure to increasing iron(III) concentration, with corresponding lifetimes; Figure S12. High resolution C1s, N 1s, and O 1s X-ray photoelectron spectra of the carbon dots before and after iron addition; Figure S13. Comparison of the carbon dot excitation spectrum and iron(III) nitrate absorption spectrum; Figure S14. Stern-Volmer plot of CDs in the presence of increasing iron concentration.

## Abbreviations

The following abbreviations are used in this manuscript:

CDs	Carbon dots
LED	Light-emitting diode
UV	Ultraviolet
XPS	X-ray photoelectron spectroscopy
FTIR-ATR	Fourier transform infrared spectroscopy attenuated total reflectance
HRTEM	High-resolution transmission electron microscopy
XRD	X-ray diffraction
ICP-MS	Inductively coupled plasma mass spectrometry
AMD	Acid mine drainage
