# Supplementary material for: A Synthetic Pathway for Producing Carbon Dots for Detecting Iron Ions Using a Fiber Optic Spectrometer
Source: Sensors (Basel). 2025 Oct 2;25(19):6066. doi: 10.3390/s25196066 (PMC12526900; doi:10.3390/s25196066)
Supplement: Supplementary file 1 [file sensors-25-06066-s001.zip › Iron_Sensor_Supporting_Information_R4.pdf]

Supporting Information

A synthetic pathway for producing carbon dots for detecting iron ions using a fiber optic spectrometer

Ariana Adkisson [1], Dean Gouramanis [2], Ki-Joong Kim [1], Ward Burgess,[1,3] Nicholas Siefert,[1] and Scott Crawford [1,*]

[1]National Energy Technology Laboratory, 626 Cochrans Mill Rd., Pittsburgh, PA USA 15236
[2]Fluid Photonics Corporation, Plainview, New York
[3]NETL Support Contractor, 626 Cochran Mill Road, Pittsburgh, Pennsylvania 15236

*Scott.Crawford@netl.doe.gov

Table of Contents:

Figure S1. TEM size distribution histogram and additional images S3
Figure S2. Representative emission spectra (left) and Stern-Volmer plot (right) for carbon dots exposed to increasing concentrations of iron(III) in 0.1 M HCl S4
Table S1. Inductively-coupled plasma mass spectrometry characterization of acid mine drainage and an AMD solids leachate in sulfuric acid…………………………………………......S4
Figure S3. Emission spectra and plot of maximum intensity vs. added iron(III) concentration for the carbon dots in a sulfuric acid AMD solids leachate to which increasing Fe(III) was added..S4
Table S2. pH and ICP-MS characterization of ion exchange column fractions of AMD solids leachate (in ppm) S5
Table S3. ICP-MS characterization of acetic acid and formic acid leachates from AMD solids (in ppm) S5
Figure S4. Emission spectra of CDs in a formic acid AMD solids leachate and in an acetic acid AMD solids leachate at different levels of dilution……………………………………………….S6
Table S4. Comparison of optical properties, synthesis, and sensing performance of iron-responsive m-phenylenediamine-derived carbon dots.. S6
Figure S5. Representative emission spectra and Stern-Volmer plot for carbon dots exposed to increasing concentrations of iron(III) in pH 1.68 buffer, measured using the portable fiber optic spectrometer. S7
Figure S6. Photographs of carbon dot-coated filter paper exposed to increasing concentrations of iron(III) nitrate (0.000001M to 1 M) under ambient and 365 nm light. S7
Figure S7. Photographs of carbon dot-coated filter paper exposed to 1M aqueous solutions of different metals commonly found in acid mine drainage and other coal utilization byproducts under ambient and 365 nm light. S8
Figure S8. Block diagram and photograph of the test strip analyzer for carbon dot-coated iron(III) test strips…………………………………………………………………………..…...S8
Figure S9. Analysis of test strip emission recorded using the Fluid Photonics Quick Strip analyzer over the course of 2 weeks under different storage conditions................................. S9

Figure S10. Absorption spectra of carbon dots in water before (blank line) and after the addition of 19 and 37 ppm iron(III) nitrate S9
Figure S11. Time resolved luminescent decays of carbon dots before and after exposure to increasing iron(III) concentration, with corresponding lifetimes. S10
Figure S12. High resolution C1s (A), N 1s (B) and O 1s (C) X-ray photoelectron spectra of the carbon dots before and after iron addition S11
Figure S13. Comparison of the carbon dot excitation spectrum and iron(III) nitrate absorption spectrum……………………………………………………………………………………….....S12
Figure S14. Stern-Volmer plot of CDs in the presence of increasing iron concentration……..S12
References S13

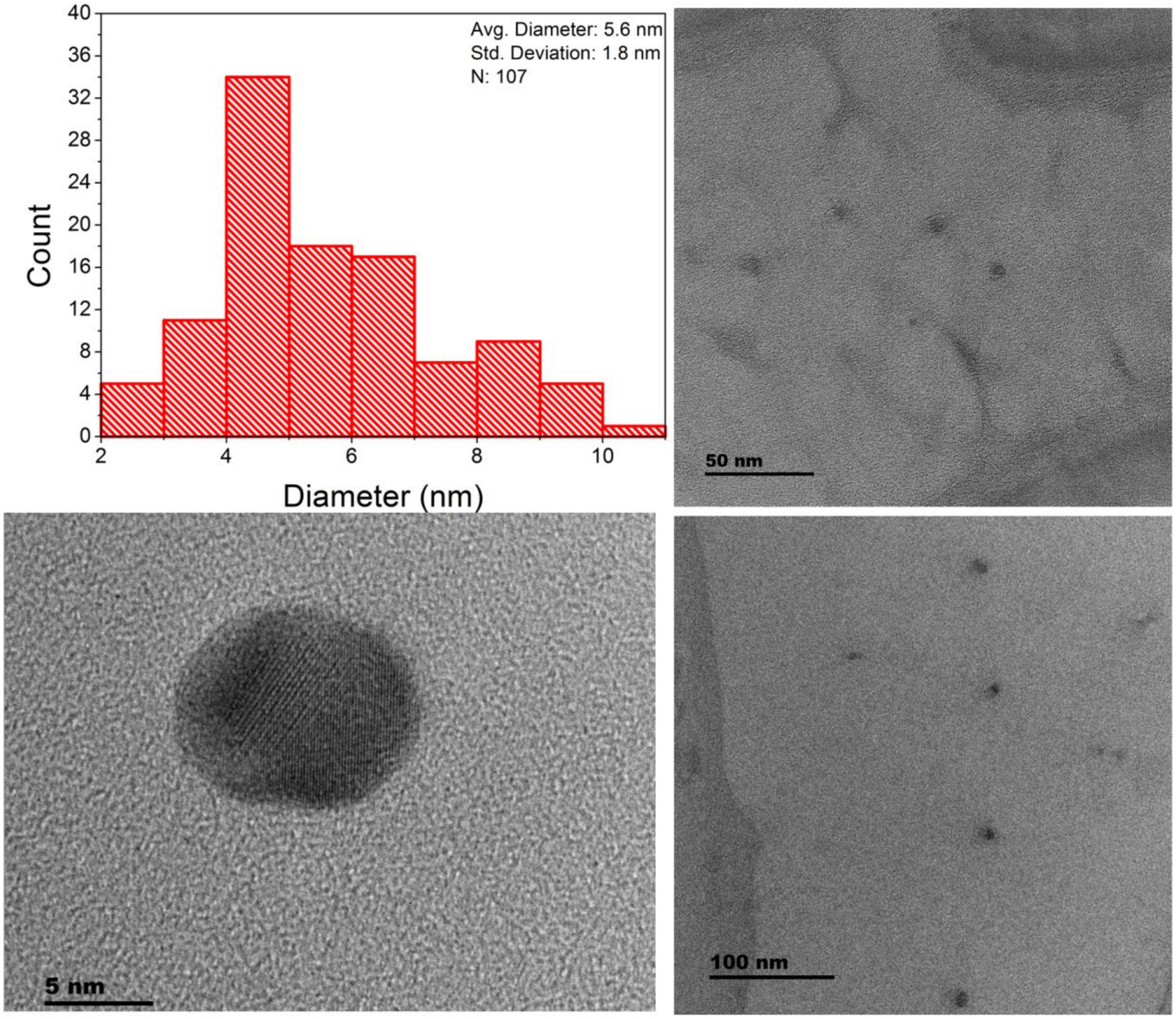

Figure S1. Size distribution histogram of the carbon dots as determined by high-resolution transmission electron microscopy (top left), along with additional TEM images at different magnifications.

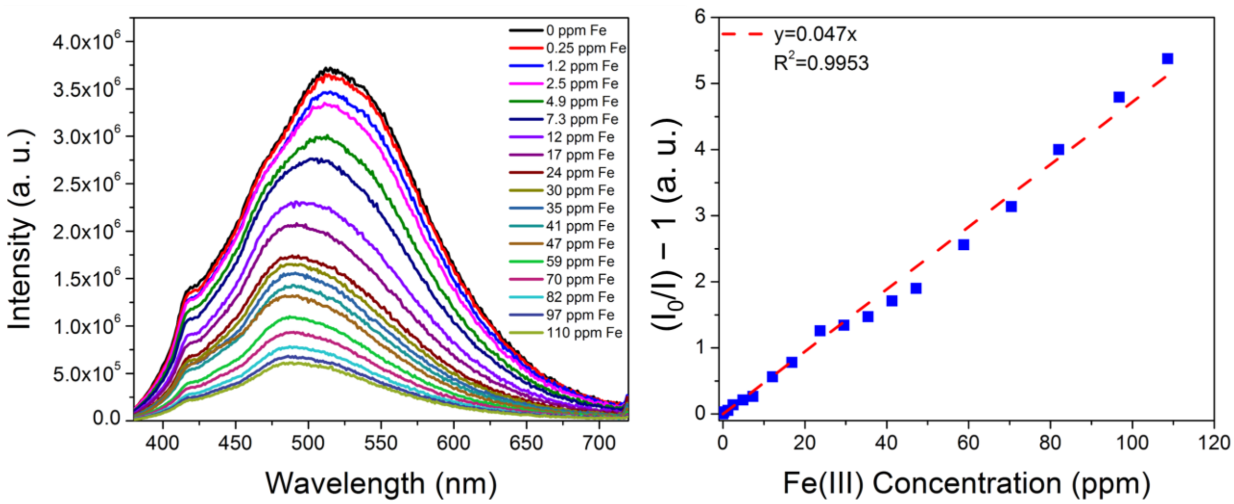

Figure S2. Representative emission spectra (left) and Stern-Volmer plot (right) for carbon dots exposed to increasing concentrations of iron(III) in 0.1 M HCl**,** measured using a commercial spectrometer. Detection limits of ~0.5 ppm iron were obtained in 0.1 M HCl, determined through three independent trials.

Table S1. Inductively coupled plasma mass spectrometry (ICP-MS) characterization of the acid mine drainage (AMD) and AMD solids leachate in sulfuric acid (after 1:20 dilution)

Metal	AMD Concentration (ppm)	AMD Solids H_2SO_4 Leachate
Al	7.5	510
Ca	67	30
Mg	47	6
Si	10.8	230
K	3.6	-
Mn	14	15
Fe	0.3	15
Zn	0.6	20
Co	0.3	0.5
Ni	0.3	4

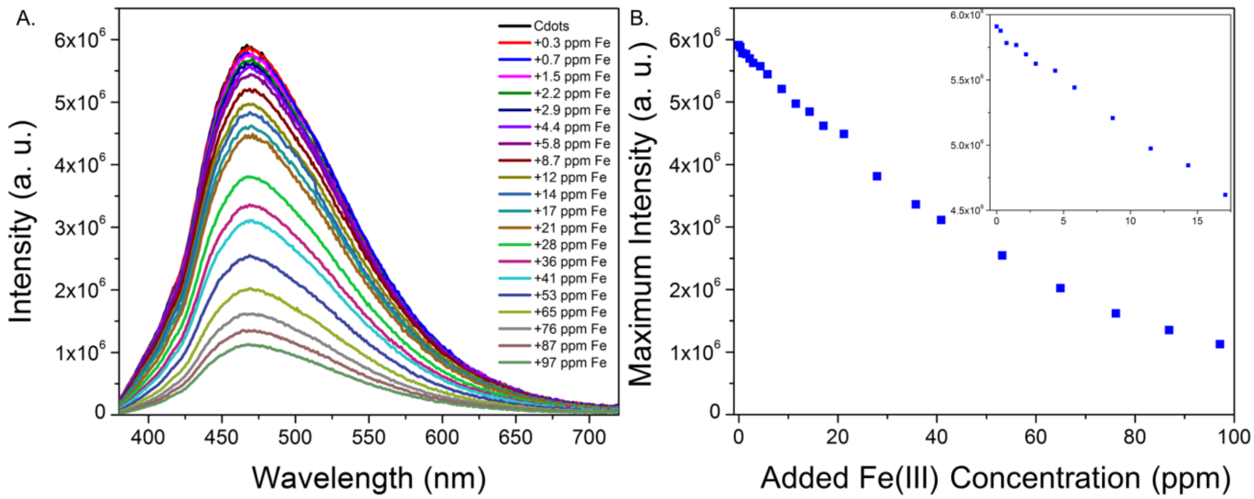

Figure S3. Emission spectrum (A) and plot of maximum intensity versus added Fe(III) concentration (B) of the carbon dots in a sulfuric acid (pH 2.7, composition in Table S1) acid mine drainage solids leachate, showing gradual quenching with increasing iron concentration. The inset zooms in on the 0-18 ppm Fe(III) range for clarity.

Table S2. pH and ICP-MS characterization of ion exchange column fractions of AMD solids leachate (in ppm)

Sample	pH	Al	Ca	Na	Mg	Si	Mn	**Fe**
A	1.18	940	1670	444	164	51	56	**259**
B	1.05	8.6	16.5	214	0.1	46	9	**2**
C	1.18	515	602	910	174	53	47	**176**
D	1.25	993	1550	451	193	51	64	**288**
E	1.31	953	1690	440	165	52	55	**256**
F	1.29	947	1660	446	165	53	56	**260**
G	1.3	969	1740	437	164	53	55	**254**

Table S3. ICP-MS characterization of acetic acid and formic acid leachates from AMD solids (in ppm)

**Metal**	**Formic Acid Leachate**	**Acetic Acid Leachate**
Al	4302	2497
Na	533	394
Mg	1877	1811
Si	4499	950
K	85	48
Ca	10664	7911
Ti	228	79
Mn	6	4
**Fe**	**1749**	**1369**
Zn	7	15
Sr	153	87

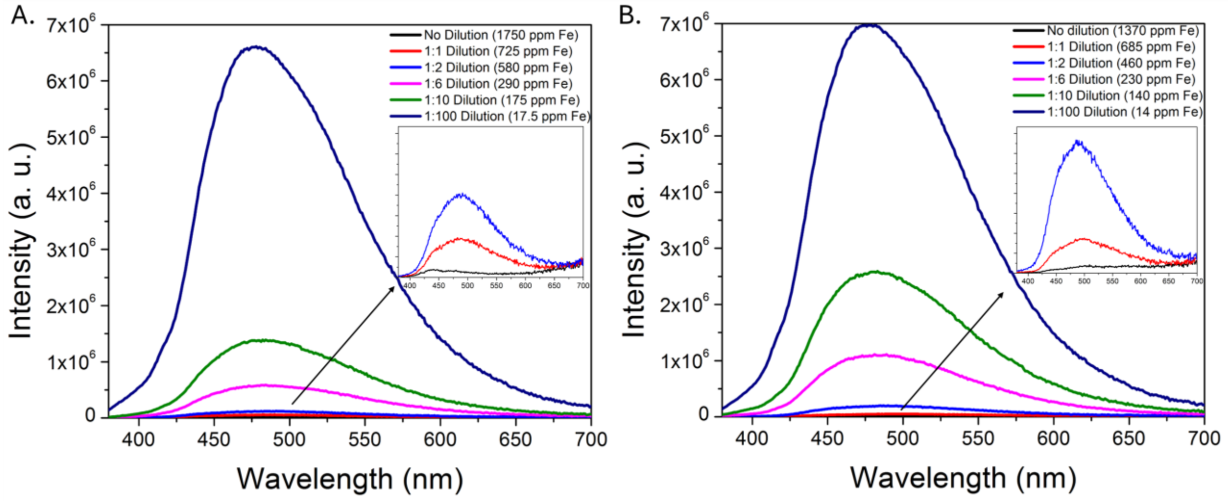

Figure S4. Emission spectra of CDs in a formic acid AMD solids leachate (A) and in an acetic acid AMD solids leachate (B) at different levels of dilution (Insets: weak emission spectra of CDs in less dilute leachates, plotted with the same intensity scale). The CDs are more emissive in the acetic acid leachate, which contains less iron, at higher dilution levels.

Table S4. Comparison of optical properties, synthesis, and sensing performance of iron-responsive m-phenylenediamine-derived carbon dots

Dopants	Synthesis Time (hr)	Synthesis Temperature (°C)	Quantum Yield (%)	LOD (ppm)	Linear Range (ppm)	pH Analyzed	# of interfering metals analyzed	Ref.
7.1%N, 0.1%P	0.5	20	9.6	0.5	~0-150	1-7	12	This Work
N (% N.R.)	3.5	180	N.R.	0.04	~0-10	7.4	8	1
~19% N	10	160	54	0.3	~0-5600	2-13	10	2
11.1%N, 9.6%P	5	200	32	0.05	~0-3.4	2-10	11	3
14.3% N	3	150	22.5	0.9	~0-55	5-11	8	4
N (% N.R.)	10	200	30.2	0.0007	~0-0.5	3	19	5
16.9% N	6	180	N.R.	0.01	~0-55	N.R.	16	6

*N.R. indicates the value wasn't reported

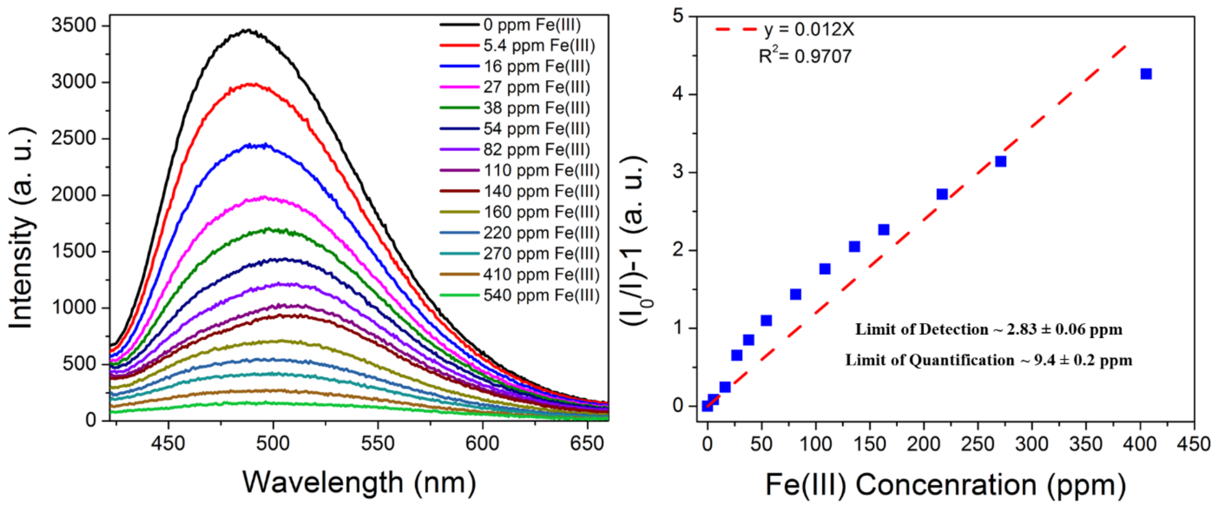

Figure S5. Representative emission spectra (left) and Stern-Volmer plot (right) for carbon dots exposed to increasing concentrations of iron(III) in pH 1.68 buffer, measured using the portable fiber optic spectrometer. Detection limits of ~3 ppm iron were obtained in pH 1.68 buffer, determined from three independent trials.

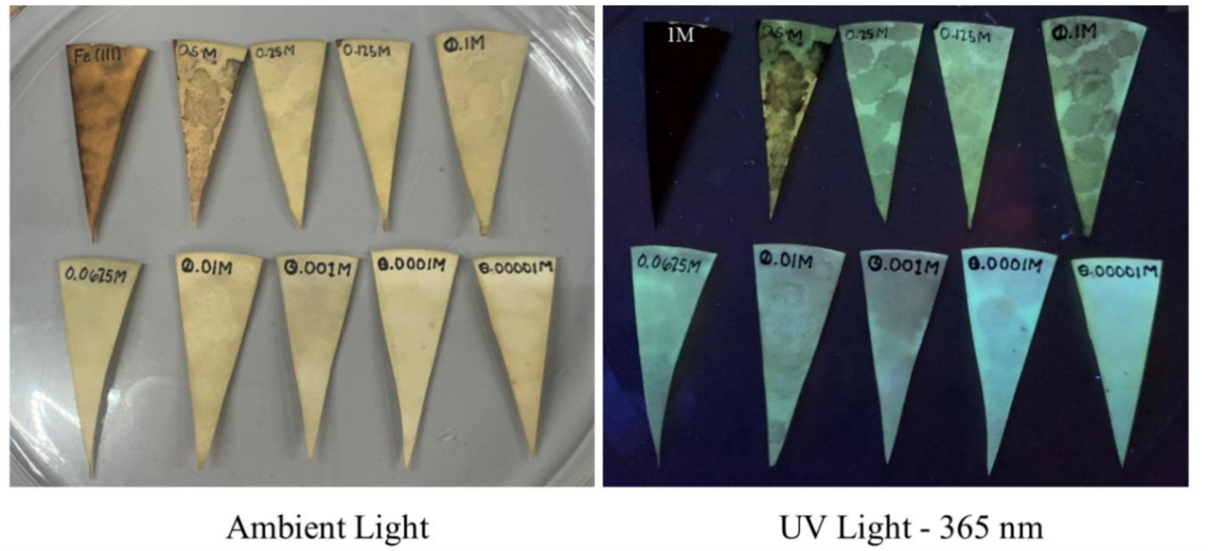

Figure S6. Photographs of carbon dot-coated filter paper exposed to increasing concentrations of iron(III) nitrate (0.000001M to 1 M) under ambient (left) and 365 nm (right) light.

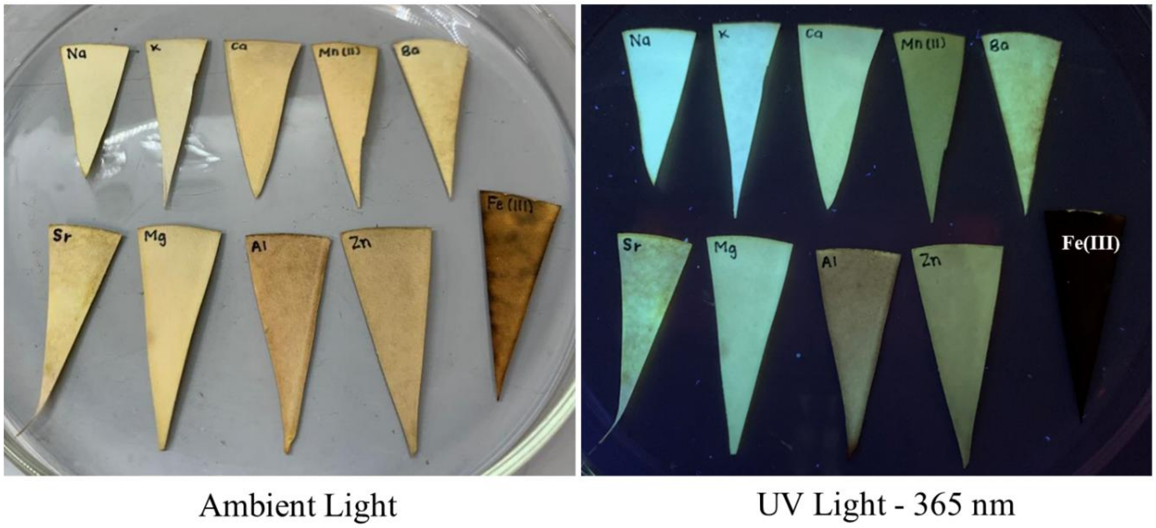

Figure S7. Photographs of carbon dot-coated filter paper exposed to 1M aqueous solutions of different metals commonly found in acid mine drainage and other coal utilization byproducts under ambient (left) and 365 nm (right) light.

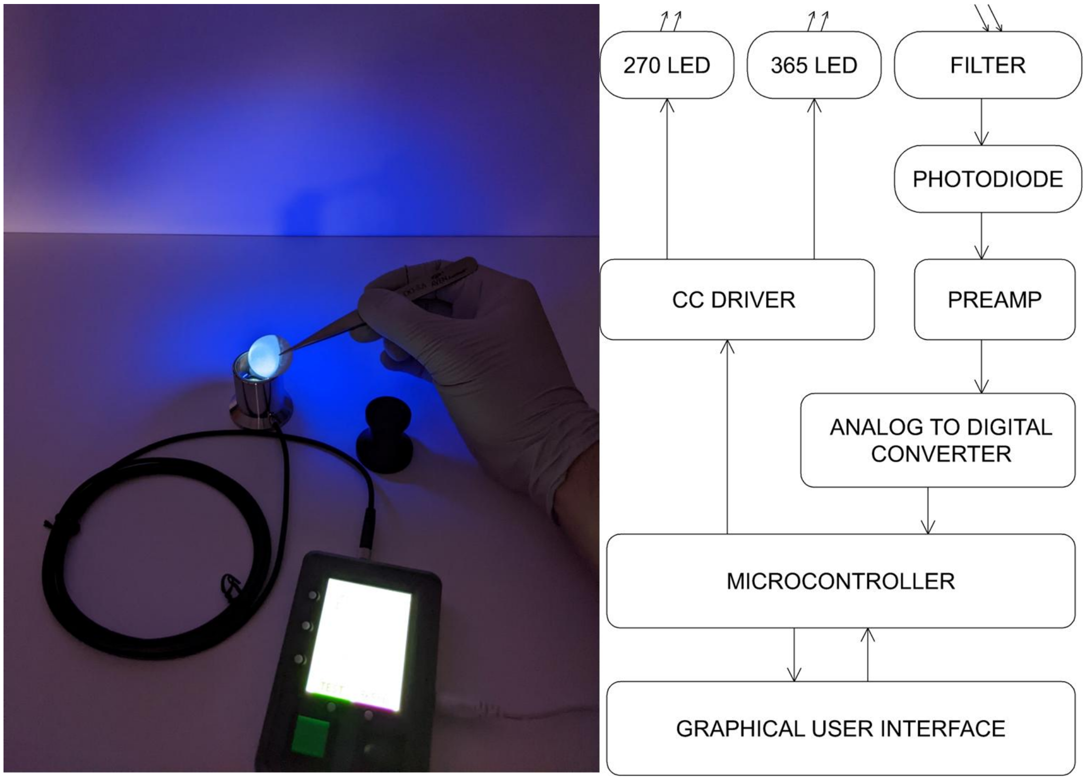

Figure S8. Block diagram (left) of the Quick Strip analyzer for quantitative detection of iron using carbon dot-coated strips. A photograph of the analyzer in action is shown (right).

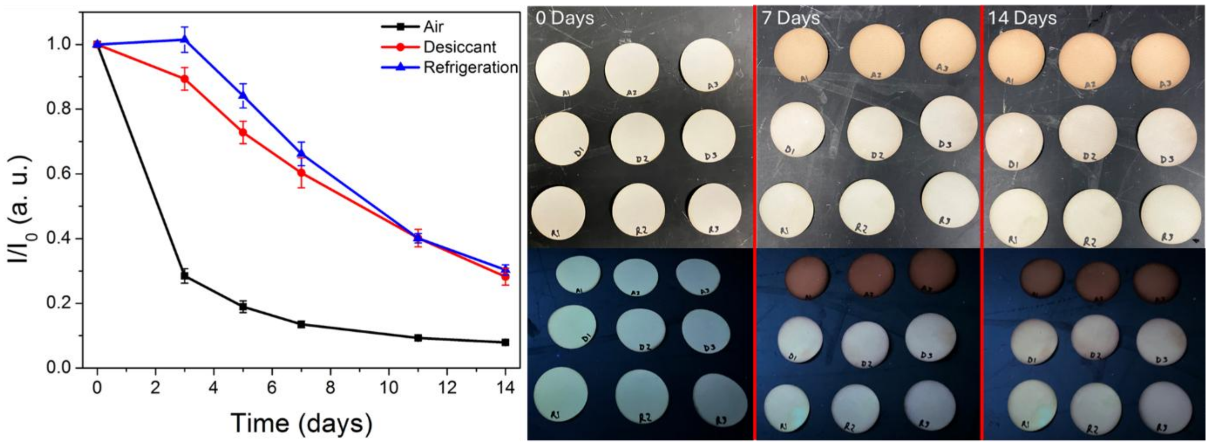

Figure S9. Analysis of test strip emission recorded using the Fluid Photonics Quick Strip analyzer over the course of 2 weeks under different storage conditions. The emission properties of test strips degrade over the course of two weeks, although storage methods such as desiccation or refrigeration improve the stability relative to the control group. Error bars denote the standard error of three independent trials. Photographs of the test strips (top row: stored in air, middle row: stored with molecular sieves, bottom row: stored in a refrigerator) under ambient and UV light are shown at 0 days, 7 days, and 14 days.

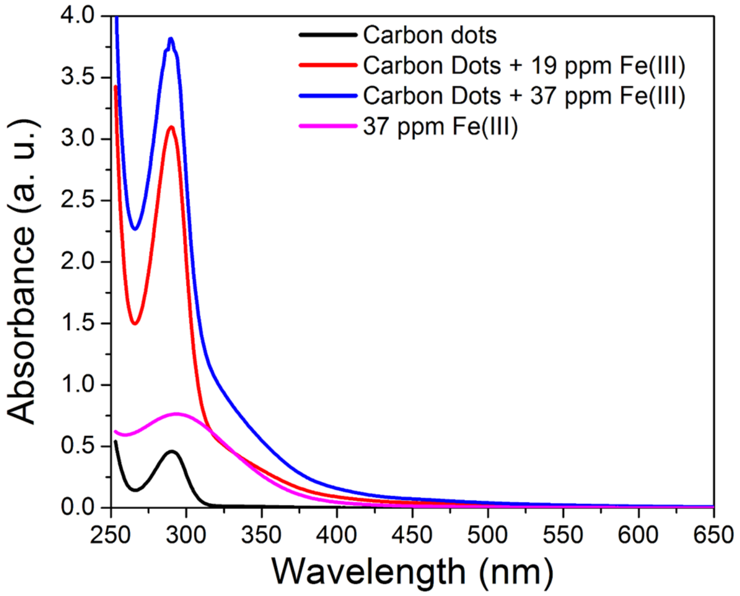

Figure S10. Absorption spectra of carbon dots in water before (blank line) and after the addition of 19 and 37 ppm iron(III) nitrate (red line and blue line, respectively). The spectrum of 37 ppm iron(III) nitrate is also shown (pink line) for comparison. The significant increase in intensity at ~290 nm of the carbon dot-iron(III) samples is consistent with complex formation between iron and functional groups on the carbon dot surface.

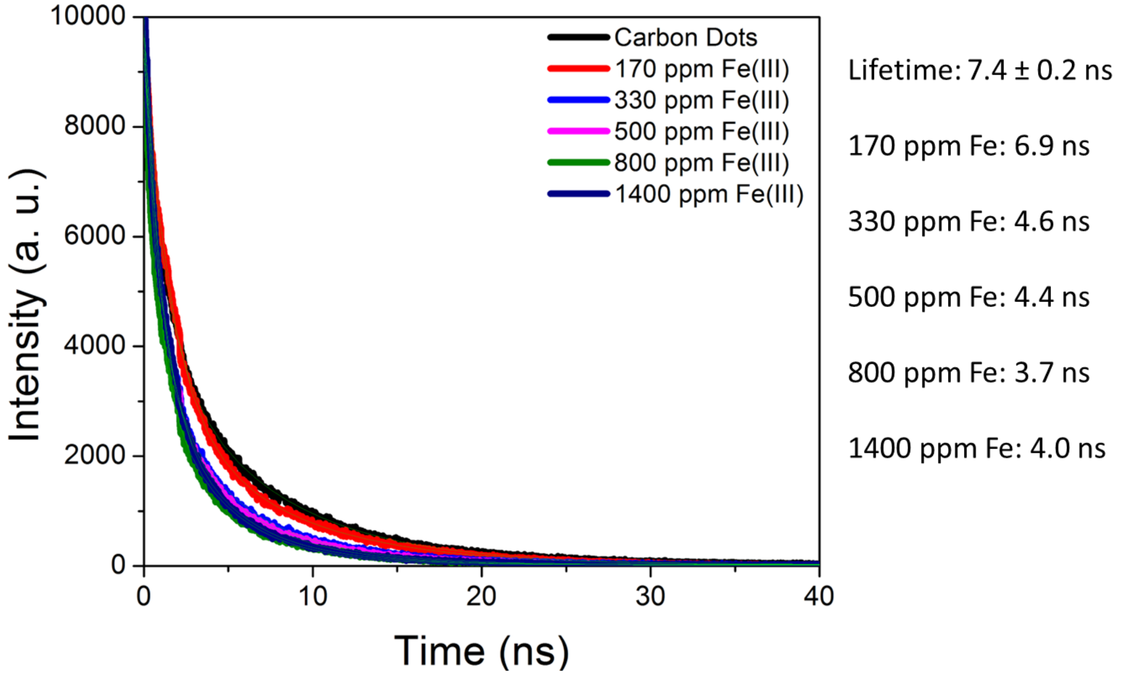

Figure S11. Time resolved luminescent decays of carbon dots before and after exposure to increasing iron(III) concentration, with corresponding lifetimes. A graduate decrease in the emission lifetime is observed as a function of increasing iron concentration.

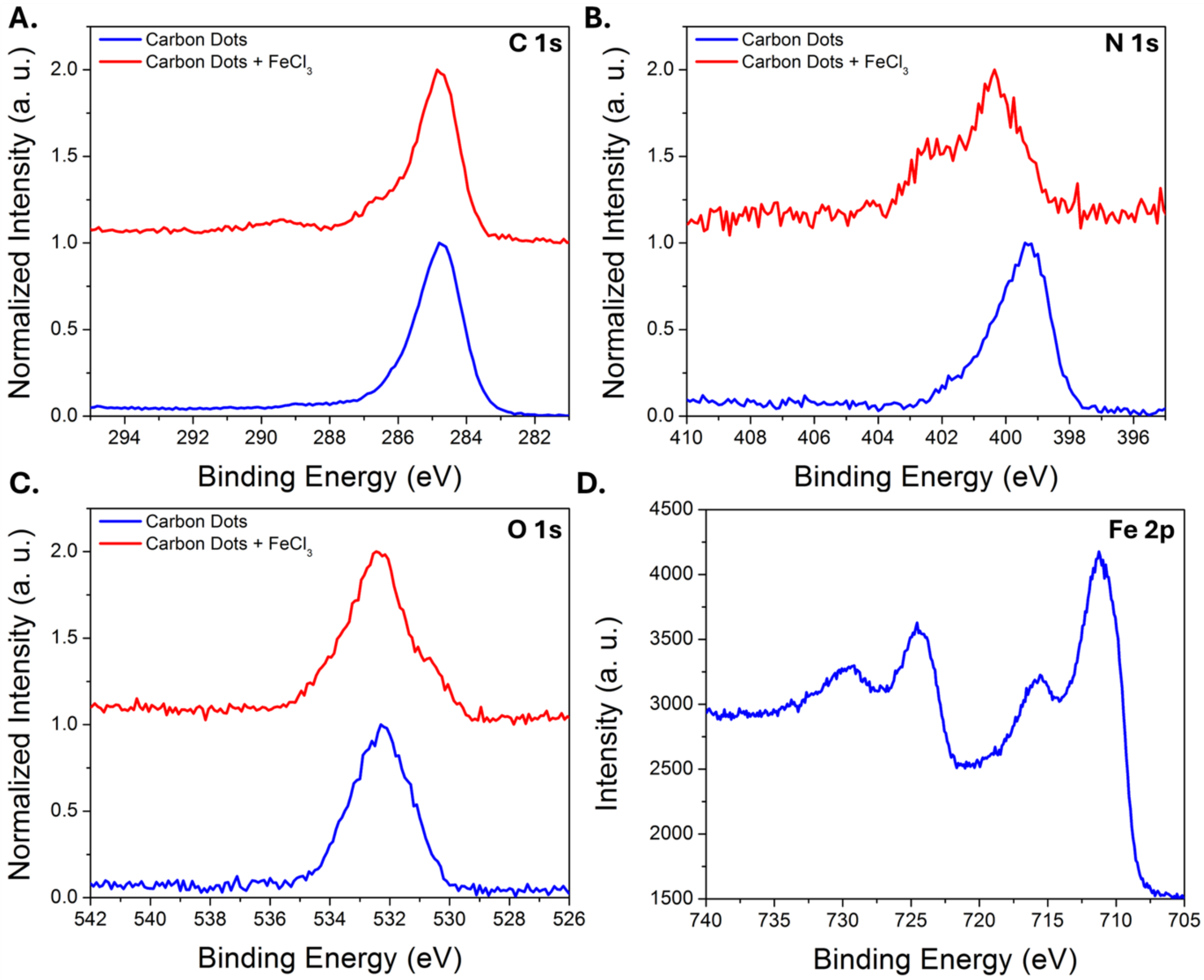

Figure S12. High resolution C1s (A), N 1s (B) and O 1s (C) X-ray photoelectron spectra of the carbon dots before and after iron addition**.** The high-resolution Fe 2p spectrum of the iron-exposed carbon dots is shown in (D).

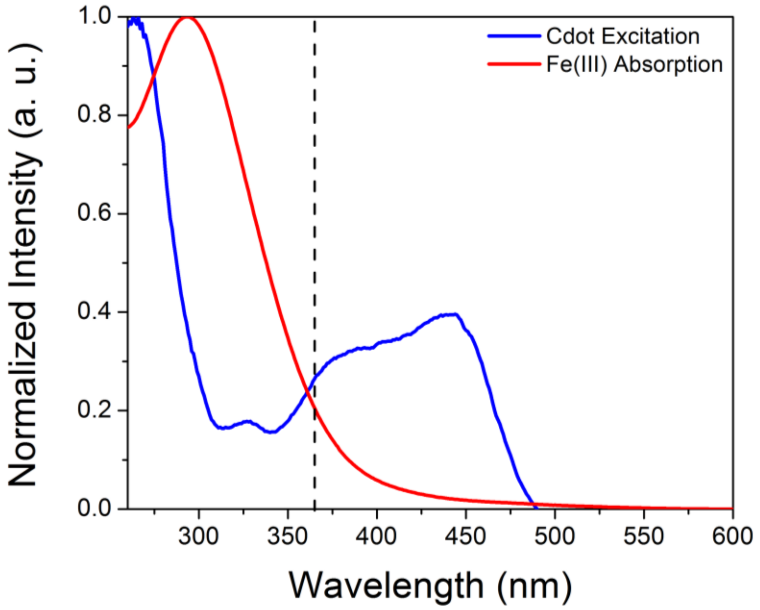

Figure S13. Comparison of the carbon dot excitation spectrum and iron(III) nitrate absorption spectrum, with a dotted line signifying the excitation wavelength used in this work (365 nm). There is partial overlap between Fe(III) absorption and the carbon dot excitation band at 365 nm, which may contribute to the quenching mechanism.

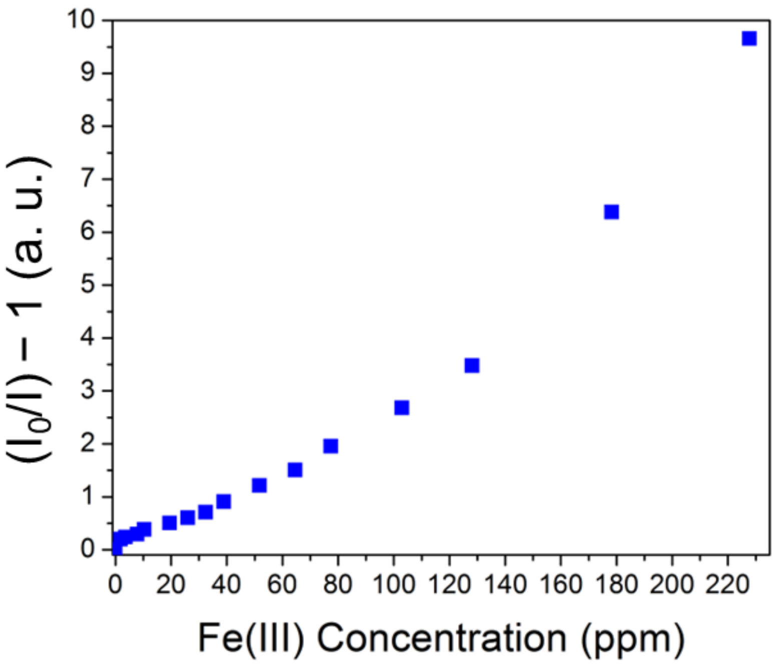

Figure S14. Stern-Volmer plot of CDs in the presence of increasing iron concentration, illustrating an upward curve deviating from linearity. This upward curving deviation from linearity is consistent with a combination of static and dynamic quenching pathways.

References

1. Fatkhutdinova, L. I.; Barhum, H.; Gerasimova, E. N.; Attrash, M.; Kolchanov, D. S.; Vazhenin, I. I.; Timin, A. S.; Ginzburg, P. and Zyuzin, M. V. *ACS Applied Nano Materials* **2023**, *6*, 23130.
2. Arkin, K.; Zheng, Y.; Hao, J.; Zhang, S. and Shang, Q. *ACS Applied Nano Materials* **2021**, *4*, 8500.
3. Li, J.; Jiao, Y.; Feng, L.; Zhong, Y.; Zuo, G.; Xie, A. and Dong, W. *Microchimica Acta* **2017**, *184*, 2933.
4. McEnroe, A.; Brunt, E.; Mosleh, N.; Yu, J.; Hailstone, R. and Sun, X. *Talanta Open* **2023**, *7*, 100236.
5. Hashemi, N. and Mousazadeh, M. H. *Opt. Mater.* **2021**, *121*, 111515.
6. Wang, Y.; Chang, Q. and Hu, S. *Sensors and Actuators B: Chemical* **2017**, *253*, 928.
